# Field and Laboratory Evaluation of Bioefficacy of an Insect Growth Regulator (Dimilin) as a Larvicide against Mosquito and Housefly Larvae

**DOI:** 10.1155/2011/394541

**Published:** 2011-09-18

**Authors:** Shandala Msangi, Ester Lyatuu, Eliningaya J. Kweka

**Affiliations:** Division of Livestock and Human Health Disease Vector Control, Tropical Pesticides Research Institute, P.O. Box 3024, Arusha, Tanzania

## Abstract

The inhibitory function of Dimilin (Diflubenzuron), mostly a chitin synthesis regulator, on the ecdysis of mosquitoes (*Anopheles gambiae* s.l., *Culex quinquefasciatus*) and housefly was evaluated in the field and in laboratory. Three formulations of Diflubenzuron were evaluated in this study: Dimilin, Wettable powder (25%), Dimilin granules (2%), and Dimilin tablets (2%). The laboratory and field evaluation used different rates of concentrations of these formulations. Generally, at higher dosages larvae developments, eggs hatchability and pupation were impossible. The development of mosquitoes was significantly higher in control while highly depressed in different dosages of treatment in both laboratory and field experiments. In houseflies, the adult population decreased sharply after treatment of their breeding sites while pupae mortality was noticed to be high in laboratory-treated samples. Dimilin could be opted as one of the choice of the larval control chemicals to be incorporated in the integrated vector control programmes in urban and rural areas.

## 1. Introduction

The mosquito *Anopheles gambiae* s.l., and *Culex quinquefasciatus* (Diptera: Culicidae) are the principal vector of malaria and filariasis in several tropical regions, respectively. Pesticide treatment has continued to be employed as the principal measure for their control [1]. However, resistance of mosquitoes to organochlorides, organophosphate, and pyrethroid pesticides have been reported in various studies [2–7]. These pesticides are frequently used for their control as well as in agriculture.

The control of mosquitoes can best be achieved through integrated vector management [8, 9]. Although control of the adult mosquitoes by using insecticides, either in indoor residual spraying or by insecticide-treated materials, are currently the most widely used strategy [10–12], the control of larvae at their breeding sites is another suitable option [9, 13]. The strategy may reduce population of adult mosquitoes by proper and selective larviciding in the breeding habitats of mosquitoes.

Larviciding involve the use of both chemical insecticides and the insect growth regulators (IGR) in controlling larvae of various insect pests [14–16]. The IGRs, unlike the chemical larvicides, are strictly arthropod-specific and environmental safe [17]. In public health, the larvicides are usually indicated for vectors or pests which tend to breed in permanent or semipermanent water bodies or places that can be identified and treated [18]. Therefore, larviciding programs can be complementary to control measures aimed at controlling malaria and other mosquito-borne diseases, nuisance mosquitoes, or other arthropods in integrated pest control programs [19].

Many countries have increased their emphasis on green revolution as an effort to increase food security for their increasing population. Although irrigation schemes may increase food production, irrigation activities usually create good aquatic breeding habitat to waterborne disease vectors like malaria and filariasis [20–23] and snails, the intermediate host of schistosomiasis [20]. Therefore, expiation of irrigated agricultural schemes expose people to higher health risks associated with vector-borne diseases [24, 25]. Efforts should, therefore, be made to incorporate waterborne vector control activities alongside all established irrigation projects. 

Here, we report results of a trial conducted to examine the effects of different concentrations of an insect growth regulator (IGRs), Diflubenzuron (Dimilin) to *An. gambiae* s.l., *Cx. Quinquefasciatus*, and housefly population in both laboratory and in the field. Our hypothesis was that exposure to IGRs would reduce the mosquitoes and houses flies emerging population density and extend duration to attain maturity.

## 2. Materials and Methods

### 2.1. The Study Site

The laboratory tests were conducted at the Tropical Pesticides Research Institute laboratory using laboratory-maintained mosquito and housefly larvae. The field trials on mosquitoes were conducted at the Lower Moshi irrigation scheme area which is well described by other studies [24, 26]. The irrigation activities in the area continue throughout the year and, therefore, provide good aquatic habitat stability throughout the year. The abundant mosquito species in the area are *Anopheles gambiae *s.l. and *Culex quinquefasciatus* with *An. funestus* as minority species [24, 26]. Field tests on houseflies were conducted in a garbage-dumping site in the Arusha urban. The studies were conducted between April and November, 2008.

### 2.2. Evaluated Products

Dimilin is an insect growth regulator belonging to the benzoyl ureas class of insecticides that inhibits the synthesis of chitin and, hence, interferes with molting; its active ingredient is Diflubenzuron, which is mainly a stomach poison and, to a lesser extent, a contact poison and acts by disturbing the molting process of all stages of larvae instars of mosquitoes and other flies [18]. The deviation from normal molting process ultimately leads to the death of the larvae. Dimilin is said to have very low toxicity to mammals, birds, fish, honey bees, and most aquatic invertebrates [27] with the exception of small crustaceans (water flees, etc.), and, hence, its effect on the environment is practically very minimal. Diflubenzuron has successfully passed the WHO's pesticide evaluation scheme for mosquito larviciding and is one of the WHOPES's recommended compounds for control of mosquito larvae, [19].

Three formulations of Dimilin were tested both in laboratory and in the field. Wettable powder (WP-25), with 250 g active ingredient per liter, were suitable for treatment of clear surface water mosquito breeding sites. Granules (GR-2), with 20 g of active ingredient per kilogram, suitable for treatment of both clear water surface and areas with grass or water plants as in rice fields. Tablets (TB-2), with 20 g active ingredient per kilogram, suitable for treatment of manholes, pits, and pit latrines, where culicine mosquitoes prefer to breed. While mosquito larvae were tested with all the 3 formulations, houseflies were tested with wettable powder only, which is the mostly recommended formulation for housefly breeding sites.

### 2.3. Evaluations of Dimilin Bioefficacy against Mosquito Larvae in the Laboratory

The Dimilin granule (GR-2) was evaluated against two species of mosquitoes, *An. gambiae *s.s. and *Cx. quinquefasciatus* from laboratory-maintained colonies. A mixture of second- and third-stage larvae were selected for the experiment as they were large enough and can easily be counted and had three to two stages before becoming adults and therefore, giving investigators enough time to make followups of their development. A plastic basin with diameter of 60 cm and depth of 15 cm was used in which 10 litres of clean rain water was put. A sensitive digital weighing machine was used to weigh 0.025 g of the granules, which was put in the basin and mixed well. This made a concentration of 0.0025 g/L. Later four serial dilutions were made, from which, these were 0.00125 g/L, 0.00625 g/L, 0.00033 g/L and 0.000156 g. All these five concentrations were evaluated against control (rain water alone). In each of these six basins, 50 second instar *An. gambiae* s.s. larvae were put immediately after dilution preparations, and their developmental stages changes were observed and recorded daily while provided with larvae feeds. Dead individuals were counted, recorded, and removed daily. Each of these basins was covered with a mosquito netting to contain the possible emerging adults. These tests were replicated three times so that in each dilution a minimum of 150 larvae of *An. gambiae* s.s. and *Cx. quinquefasciatus* could be evaluated. The testing room was maintained at 27-28°C and 75–80% relative humidity. 

In Dimilin's wettable powder (WP-25), the dilutions and all conditions of testing were exactly the same as described for testing Dimilin granules above. Laboratory-reared larvae of *An. gambiae* s.s. and *Cx. quinquefasciatus* were used.

In Dimilin Tablets (TB-2), laboratory-maintained mosquito larvae of both *An. gambiae* s.s. and *Cx. quinquefasciatus* were used for the test. Manufactures recommended rate of 25 tb/m^3^ was used in which one tablet was dissolved into 40 litres of rain water. Five litres of the solution was then put into each of 5 plastic basins prepared for the tests. One basin was used as our control in which only 5 litres of rain water was put. Fifty larvae, mixtures of second and third stages of *An. gambiae* s.s., were then put in each of the six basins provided with larval feeds and observed for development daily. Dead individuals were counted and removed from the basins daily until all larvae died or developed to adults. *Cx. quinquefasciatus* larvae were also tested in a similar way. 

### 2.4. Residual Effectiveness of Dimilin Formulations and Effects on Egg Hatchability

#### 2.4.1. Dimilin's Granules and Wettable Powder

The lowest and highest concentrations were used to test the effect of Dimilin on the hatchability of mosquito eggs in which one filter paper with mosquito eggs was put in each of three plates with water treated with Dimilin at 0.0025 g/L (highest) and 0.000156 g/L (lowest) and a control. To test for the residual effectiveness of the product in water, an average of 50 second instar larvae were introduced into another set of the treated plates and control at days 7, 14, and 21 after dilutions and larval development monitored.

#### 2.4.2. Dimilin Tablets

In a separate experiment, *An. gambiae* s.s. eggs in filter paper was put in a plate with rain water treated with Dimilin tablets at a rate of 25 tb/m^3^ to test the effect of Dimilin on mosquito eggs. Another filter paper with eggs was put in clean rain water plate as a control. To assess the residual activity of Dimilin tablet in water, 50 second stage larvae of *Anopheles gambiae *s.s. were inoculated into similar treated basins on days 7, 14, and 21 and their development monitored.

#### 2.4.3. Houseflies Laboratory Tests

Two laboratory tests on houseflies were conducted. In the first experiment, laboratory-maintained houseflies (*Musca domestica*) were allowed to lay their eggs in wet layer's mash in plates placed inside netted cage. The emerged larvae (maggots) were then used for the tests in their third instar, in which 0.025 g of Dimilin powder was mixed with 5 litres of water (0.005 g/L) and used to wet 0.5 kg of layer's mash in plates. About 150 third instar maggots were then introduced into this treated substrate as their feed. The plate was then put inside a large cage covered by mosquito netting. Two replicates were made. In another plate, a higher concentration of Dimilin powder of 0.025 g mixed with 2.5 litres of water (0.01 g/L) was used. A control experiment was set aside in which the layer mash was made wet by clean rain water and 150 maggots put. A close observation was made on the development of the maggots and recorded. 

In the second experiment, flies were made to lay their eggs in a treated layers-mash substrate, in which 0.025 g of Dimilin powder was mixed with 2.5 litres of rain water and used to wet 0.5 kg of layer's mash in plate. The plate was then put inside a large cage with adult houseflies for them to lay their eggs for two days on a treated substrate. After two days, the plate was then transferred to another empty cage with no flies and observation made on development of larvae from the eggs.

### 2.5. Field Bioefficacy Evaluation of Different Dimilin Formulations against Mosquito Larvae

#### 2.5.1. Granular Dimilin

A survey to identify potential breeding sites was conducted in rice farm plots in Lower Moshi. Four rice farm plots flooded with water were identified and selected for the study, and the owners contacted. The four farms were identified in Pasua (2 farms) Block 5 (1 farm) and Kikwateni corner (2 farms). One of these sites at Pasua farms and one at Kikwateni farms were made control plots while the rest 3 were treated with Dimilin. The owners were requested to allow sampling to be done in their plots. They were requested not to allow water in or out of their plots through the irrigation canals before the end of sampling period. The planted rice in these sites were 15–30 cm tall. Pretreatment sampling was done using a 350 mLs standard dipper in different positions of the flooded rice plots randomly as described elsewhere [28]. Water was then sieved using a standard sieve described in WHO's entomological manual [28] to collect larvae and pupae. They were then identified species-wise using morphological identification key [29], thereafter, counted, recorded, and returned back to the sampled site. Treatment with Dimilin granules was done at a rate of 3000 g/h by throwing the granules evenly into all areas of the rice plot. 

Posttreatment sampling was done in a similar way as for pretreatment and was done after every other day for 12 days, when existed mosquito eggs or larvae at first instars at the time of treatment were expected to have pupated. After day 12, sampling was done after every 7 days until day 30 to monitor residual activity of the granules.

#### 2.5.2. Dimilin's Wettable Powder

For Dimilin WP-25, a survey was made in the study area, and four different breeding sites were chosen. These were closed-system water bodies, large ponds with clear water surfaces, or rice field with very young plants. They were identified as Usagara floods (1 site) Kikwateni farms (3 sites), and Mbugani ponds (1 site). Two of the sites at Kikwateni farms were made controls while all the remaining 3 sites were used for treatments with the Dimilin's wettable powder. Pretreatment sampling was made just before the actual treatment. A 350 mLs dipper was used in sampling in which the dipper was dipped randomly in different positions of the experimental rice plot. Standard sieve was used to sieve/decant the water from the bucket to leave behind mosquito larvae or pupae. The collected pupae and larvae were identified species-wise, counted, recorded, and returned back to their original site. Treatment was then done by application of Dimilin powder at a rate of 300 g/h. Posttreatment sampling was done in the same way as for pretreatment sampling. This was done every other day until day 12 after treatment, when existed eggs or first larvae instars at the time of treatment were expected to have pupated. After day 12, sampling was done after every 7 days until day 30 to monitor for the residual activity of the powder.

#### 2.5.3. Dimilin Tablets

For the Dimilin tablets (TB-2) evaluation was done in abandoned water pits, which provide efficient breeding habitats of mosquitoes. Many of these pits had dirty water of varying degrees. The selected pits were identified as Pits No. 1 to 6. The volume of water contained in each of the pits was estimated by measuring the width, length, and depths of the part occupied by the water. Pretreatment sampling was done by using standard dipper (350 mLs) for dipping in each selected habitat. Pupae and larvae were counted. Treatment with Dimilin tablets was then done at a rate of 25 tabs/m^3^ to pits 1–4, while pits 5 and 6 were left as controls. Posttreatment sampling was done in a similar way as for pretreatment sampling and was done after every other day until day 14. Residual activity of the tablets was assessed by continued sampling after every 7 days, until day 30. There were no anopheline larvae in the pits.

## 3. Data Collection and Analysis

Data were collected from both laboratory and field experiments in each evaluated compound of Dimilin. Data were entered in Microsoft access twice for validation. Statistical analysis was done using SPSS software (Version 15.0 for Windows, SPSS Inc., Chicago, Ill, USA). The number of larvae between control and treatment in each compound were compared using the samples Student's *t*-test. Odds ratios (OR) and 95% confidence intervals (CI) were calculated using website-based contingency table (http://faculty.vassar.edu/lowry/odds2x2.html) to assess the impact of treatment in habitats in the risk of adult vector abundance.

## 4. Results

### 4.1. Laboratory Test Results of the Three Formulations on Mosquito Larvae

A total of 750 *An. gambiae *s.s. and the same for *Cx. quinquefasciatus* larvae were tested in both wettable powder and the granular formulations. In Dimilin granules, some of the treated larvae managed to develop to the fourth instars. There was no pupae emerged, and this was observed in all concentrations. On the other hand, 96% of *An. gambiae* s.s. and 99% of *Cx. quinquefasciatus *pupae in the control emerged. In both formulations, the mortality of *An. gambiae *s.s. larvae was observed to start 24 hrs after treatment, and it was complete in 6-7 days time. In culicines mortality started 48 hrs after treatment, and the highest concentrations gave 100% mortality within 7-8 days while the lowest gave 100% mortality after 11 days. Most larvae died while in the third and fourth instars. There were no recorded pupae in the treated basins. In the wettable powder tests, the control basin gave 98% emergence of *An. gambiae *s.s., and 100% *Cx. quinquefasciatus* pupae were recorded, with all pupae developing to adults. The survival and development of larvae in treatment was significantly lower as compared to the control (*t* = −3.003, df = 8, *P* = 0.017). In Dimilin tablets (TB-2), a total of 250 *An. gambiae* s.s. and 250 *Culex quinquefasciatus* larvae were tested in a single concentration of 0.05 g/L. Mortality in both species, started in 48 hrs after treatment. In *An. gambiae* s.s. basins, mortality of all 250 tested larvae was complete in 5 days after introduction while in *Cx. quinquefasciatu*s larvae mortality was complete in 8 days time. Mortality of larvae occurred while the larvae were either at third or fourth instars, and there were no pupae emerged recorded. In control basin, a 100% pupae emergence was recorded in *An. gambiae* s.s. and 99% in *Cx. quinquefasciatus* larvae. The difference between control and treatment was significantly different statistically (*t* = −4.21, df = 1, *P* = 0.021).

### 4.2. Laboratory Test Results on Housefly Larvae

Dimilin powder at 0.005 g/L was able to suppress emergence of adult by 50% and at 0.01 g/L; Dimilin achieved 82% suppression of adult emergence (Table 1) in the basins where larvae (maggots) were put in a treated substrate. In the second experiment in which flies were made to lay their eggs in a treated substrate, emergence of adult was suppressed by above 91% (Table 2) in comparison to the control. The Dimilin powder treatment was 3.2 times suppressive in emerging flies than control which was statistically different (OR 3.25, 95% CI 1.69–6.24, *P* = 0.0004). The 0.01 g/L concentration of Dimilin powder achieved 3.9 times more suppression of the adult emergence than control area which was statistically significance (OR 3.97, 95% CI 1.59–9.89, *P* = 0.0016).

### 4.3. Field Bioefficacy Evaluation Results for the Three Formulations

#### 4.3.1. Dimilin Granules

**Figure 1:**
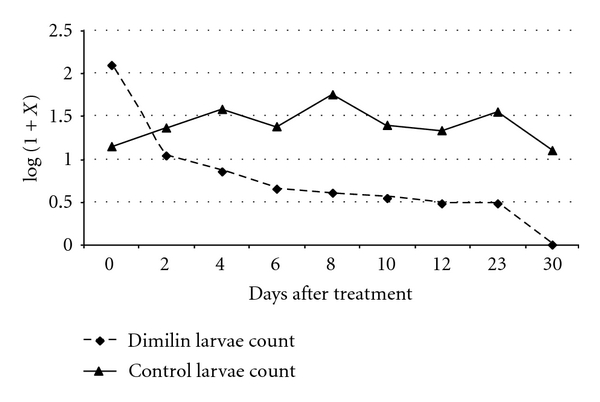
Mean anopheline larvae count in Dimilin granules-treated rice field.

**Figure 2:**
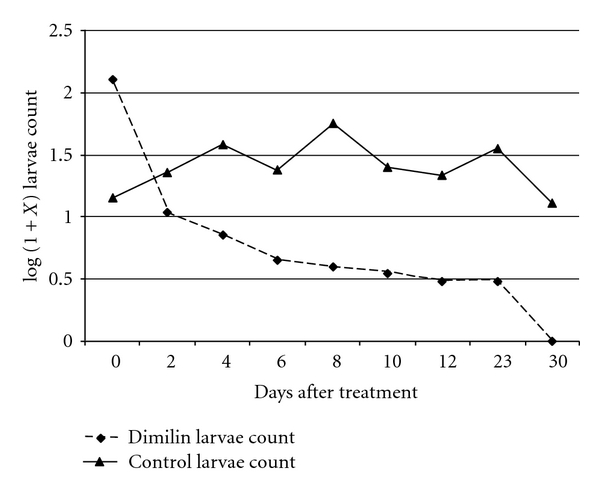
Mean culicine larvae count in Dimilin granules-treated rice plots.

anopheline and culicine larvae were the main mosquito species found in the study area. When compared with the untreated control plots, the number of larvae and pupae collected from Dimilin granules-treated plots continued to decline for all stages for both species, except for stage I in which the number recorded showed no specific trend. The total larvae population trend for *An. gambiae *s.l. is shown in Figure 1 and *Cx. quinquefasciatus* (Figure 2). In the treated plots, there was a continued overall decline of larvae population as from the treatment day for both species of mosquitoes. In the control field plots, there was a fluctuation of the larvae populations count, and, on day 30, anophelines larvae counts were almost the same and comparable to the baseline count while culicine larvae counts were higher than the baseline count.

#### 4.3.2. Dimilin's Wettable Powder

**Figure 3:**
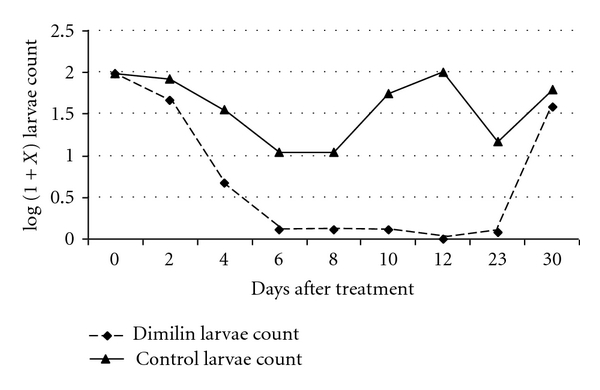
Mean anopheline larvae count in Dimilin powder-treated sites over time.

In plots where Dimilin powder was applied, with the exception of first larvae instars, all other stages showed a continuous decrease in their number from day 2 after treatment in the treated sites as compared to the control.  Figure 3 shows the overall anopheline larvae population trend over time after treatment. Culicine larvae population showed a similar trend. Except for first instar larvae, there were almost no or very few other larvae stages collected in the treated plots after 1 week. The larvae population of all stages in treated areas started to build up after 3 weeks. The control plots larvae population showed up and down trend, and, after a month, the population was almost similar to the recorded baseline pretreatment count.

#### 4.3.3. Dimilin Tablets

**Figure 4:**
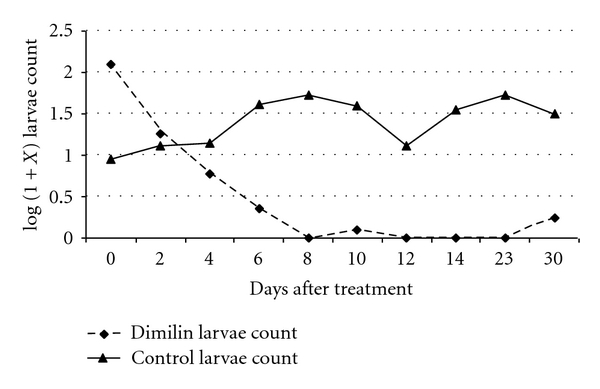
Mean culicine larvae count over time in Dimilin Tablet treated pits.

Generally, in tablets-treated pits, there was a continued reduction of larvae/pupae counts from treatment day (day 0) to day 30 in all stages. The exception to this trend was shown by stage 1 larvae in which a general increase of larvae population was recorded.  Figure 4 gives the overall population count trend of culicine larvae in the treated pits. Except for stage 1, there were not any other larvae collected in treated pits after 1 week. Culicine larvae started to develop slowly 3 weeks after treatment. In the control pit, larvae of all stages continued to be collected throughout the sampling period of one month, and, on day 30, more larvae were collected than baseline counts (day 0).

#### 4.3.4. Field Trial for Houseflies

Field trial was conducted using a garbage-dumping site around Ngaramtoni Township about 10 Km North of Arusha city where houseflies were found to breed in garbage damp. Emergence trap was set up with a dimension of (5 × 5 × 2.5) M. Three cloth stripes, smeared with a strong sticky material, were hanged diagonally in each emergent trap to attract flies to rest on them. The three cloth strings were removed after every 3 days and replaced with new ones. Flies caught at the sticky stripes were identified and counted. After 9 days with 3 samplings, treatment with Dimilin (WP-25) was done by a thorough spraying of the garbage dump to make the place just wet. The posttreatment sampling continued for another period of 21 days with 7 samplings. Due to unavailability of a similar site, the pretreatment sampling acted as its own control data. The untreated period was 8.10 more productive than treated period and statistically significant (*P* = 0.0001).

### 4.4. Eggs Hatchability and Residual Effectiveness Tests Results

#### 4.4.1. Dimilin Granules

Hatchability test on Dimilin granules showed that there was a normal hatching of eggs, both in the control and at the lowest concentration (0.000156 g/L) plates. However, all larvae obtained from the lowest concentration plate died while still in stage1, while those from the control developed to adults. There was completely no hatching in the highest concentration. The higher concentration maintained 100% mortality of larvae even after day 21. However, there was development of larvae to pupae of about 44% from the lowest concentration on those larvae inoculated on day 21. The development stopped at this stage. The control gave 100% pupae up to day 21 which developed to adults.

#### 4.4.2. Dimilin's Wettable Powder

Test on the eggs showed no hatching of eggs in the highest concentration plate. The lowest concentration showed normal hatching, similar to that in the control plate. However, larvae from the control plate developed to adults while those from lowest concentration died while in stage 1. Dimilin powder showed long residual activity up to day 21 for all concentrations, giving 100% mortality within 7 days after inoculation.

#### 4.4.3. Dimilin Tablets (TB-2)

At the tested concentrations, there was not completely any larva hatching from the eggs in the treatment plate. From the control plate, there was a normal hatching of larvae, which developed to adults. Dimilin tablets showed good residual activity for up to the second week only. Larvae inoculated on the third week (Day 21) showed stagnation in development and took over 12 days for complete mortality after inoculation.

#### 4.4.4. Housesflies Field Trial Results

**Figure 5:**
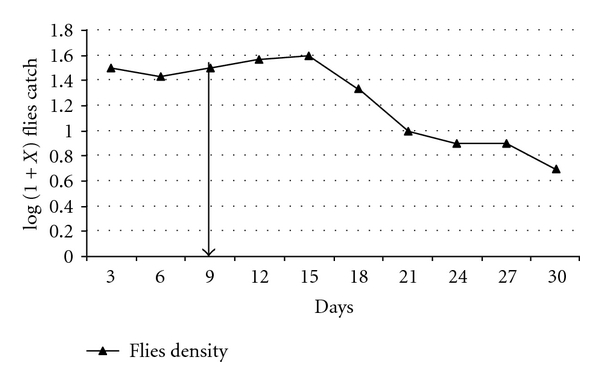
Mean housefly count over time. *Note*. The arrow marks the treatment day.

Most of the caught flies were *Musca domestica*, the common housefly. Others in low numbers were the green fly (*Lucilia* spp.) and few others. Only *M. domestica* flies were included in the counts.  Figure 5 shows the houseflies population trend before and after treatment. There was no noticeable immediate effect on the fly population after treatment. The number kept on increasing for about a week after treatment when the number started to drop down dramatically.

## 5. Discussions

Exposures to different concentrations of Dimilin Diflubenzuron have shown high rate of mortality of the larvae (*An. gambiae* s.s. and *Cx. quinquefasciatus*) as well as housefly larvae. These IGRs have produced distinct effects on larval growth of different species of mosquitoes, for example, in *An. gambiae *s.l., [30] and *A. aegypti* [18]. Negative impact of Dimilin in the growth and development of *A aegypti* larvae population have also been reported [15]. 

In this work, Dimilin has shown to be effective in controlling the larvae of the two most important mosquito species, *Anopheles gambiae*, the most important malaria vector in Africa [14, 31] and *Culex quinquefasciatus*, a vector of filariasis and an important biting nuisance mosquito species [5]. It has also been shown to be effective in controlling the larvae of houseflies, which are linked with mechanical transmission of a number of diseases including cholera and trachoma to human [32]. The effectiveness against mosquito larvae has been demonstrated even with very low concentration of Diflubenzuron, the active ingredient of the product. Our laboratory and field results show that this product is effective against all larvae stages of mosquitoes as has been found elsewhere [18, 30, 33]. However, in the field Dimilin evaluations, the number of first larvae instar in many treated sites in the field appears to be not affected as the population kept on increasing throughout the study period in treated habitats. This could be an indication that the hatching of eggs to first larvae instar was not affected by these formulations. However, this high number of stage I never reached stage II. Laboratory tests on egg hatchability indicated that, at higher concentrations, Dimilin is ovicidal and inhibit hatching of eggs, and, at lower concentration, the eggs hatch but die while still in first larvae instars which was similar to previous findings[34, 35]. From results of this work, it is, therefore, evident that, at the recommended application rate in the field, Dimilin is effective enough against all stages of larvae but not on eggs, allowing normal hatching of eggs to stage I, which thereafter dies.

Although some few larvae and pupae of both anopheline and culicines continued to be collected throughout the study period in the treated plots, there was a significant overall decrease of larvae and pupae after treatment. The population increase of larvae in 3 weeks after treatment in most treated areas in the field is an indication that, the residual activity of Dimilin is about 3 weeks and, therefore, retreatment need to be within 3-4 weeks. This observation was also supported by our laboratory results on the residual activity tests in which effectiveness of some concentrations decreased sharply in the third week. In our laboratory tests, it took 2-3 days more for all 50 *Cx. quinquefasciatus* larvae in each test to die as compared to *An. gambiae *s.s. This observation implies that culicine larvae are probably more tolerant to Diflubenzuron than anopheline* larvae*. 

The wettable powder formulation had promising effectiveness against houseflies, both in the laboratory and field. These findings have been consistent to previously reported efficacy of Dimilin WP in other areas against *Musca domestica* [9]. The continued increase of fly population for about a week, even after treatment, indicate the probability of the product to have no effect on adult flies, in which the monitoring and sampling was based but, rather, its effect was on the larvae (maggot) [9]. Its effect on larvae was reflected by decrease in the caught adults after a week.

Diflubenzuron is known to be effective as a spray against multiresistant fly population [32] and is one of compounds recommended for control of mosquito larvae [19]. It has a selective activity and is safe to fish and most aquatic invertebrates, low toxicity to human has and is safe to environment and nontarget organism [27, 36, 37]. Due to this peculiar characteristic of Diflubenzuron, Dimilin could be considered to be ideal for integrated pest management programmes.

Many developing countries lack linkage between the agricultural and health sector activities to jointly address the problem of increased health risk due to implementation of green revolution [25]. In irrigation scheme areas, where irrigation activities create favorable environment for breeding of mosquitoes, vector control activities could be incorporated in irrigation programs to reduce malaria vectors in such areas [38]. This has shown to be successful in other areas such as in schistosomiasis control [21, 22, 39]. It is of paramount importance that the policy of increasing irrigation agriculture should also consider controlling the resulting increased stable aquatic breeding habitats of mosquitoes and other vectors, as an intersectoral project, involving all relevant sectors. In urban areas where removal of garbage and domestic wastes is a problem in most towns, Dimilin could be used to spray in such garbage to control houseflies and thereby reducing such diseases linked to houseflies like diarrhea diseases in areas of higher densities. 

Based on result of different formulations of Dimilin tested in this study, the powder (WP-25), the granules effervescent (GR-2), and tablet effervescent (TB-2), we recommend the use of these three formulations in controlling mosquitoes especially in irrigation scheme areas, pit latrines, and other water bodies as well as control of houseflies in garbage and other waste-dumping sites.

## Figures and Tables

**Table 1:** Effect of Dimilin (WP) in development of housefly larvae (maggots).

Test	Dimilin treatment	No. of maggots tested	Emerged pupae from maggots (percentage)	Emerged adults from pupae	OR (95% CI)	*P* value
1	0.005 g/L	300	55 (18.3%)	12 (21.8%)	3.25 (1.69–6.24)	0.0004
	Control	300	254 (84.6%)	180 (70.8%)		
2	0.01 g/L	150	29 (19.3%)	0 (0%)	3.97 (1.59–9.89)	0.0016
	Control	150	140 (93.4%)	115 (82%)		

**Table 2:** Effect of Dimilin (WP-) treated substrate on housefly eggs and larvae development.

Dimilin	Emerged maggots	Emerged pupae	Emerged adults	OR (95% CI)	*P* value
0.01 g/L	850	52 (6%)	1 (2%)	8.11 (3.46–19.01)	*P* < 0.0001
Control	775	650 (83.9 %)	608 (93.5)		
